# 1,3-Bis[(3-allyl­imidazol-3-ium-1-yl)meth­yl]benzene bis­(hexa­fluoridophosphate)

**DOI:** 10.1107/S1600536810050683

**Published:** 2010-12-11

**Authors:** Rosenani A. Haque, Mohammed Z. Ghdhayeb, Hassan H. Abdallah, Ching Kheng Quah, Hoong-Kun Fun

**Affiliations:** a School of Chemical Sciences, Universiti Sains Malaysia, 11800 USM, Penang, Malaysia; b X-ray Crystallography Unit, School of Physics, Universiti Sains Malaysia, 11800 USM, Penang, Malaysia

## Abstract

In the title compound, C_20_H_24_N_4_^2+^·2PF_6_^−^, the ethene and 3-allyl­imidazolium moieties of the cation are disordered over two positions with refined site occupancies of 0.664 (19):0.336 (19) and 0.784 (7):0.216 (7), respectively, whereas four F atoms of one hexa­fluoridophosphate anion and all atoms in the other hexa­fluoridophosphate anion are disordered over two positions with refined site occupancies of 0.764 (5):0.2365) and 0.847 (9):0.153 (9), respectively. The benzene ring is inclined at angles of 78.2 (3), 81.3 (4) and 73.9 (12)° with the 1*H*-imidazol-3-ium ring and the major and minor components of the disordered 1*H*-imidazol-3-ium ring, respectively. In the crystal, the hexa­fluoridophosphate anions link the cations into two-dimensional networks parallel to (001) *via* inter­molecular C—H⋯F hydrogen bonds. The crystal structure is further consolidated by π–π [centroid–centroid distance = 3.672 (3) Å] and C—H⋯π inter­actions.

## Related literature

For general background to and the biological activity of carbene derivatives, see: Yang & Nolan (2001 ▶); Böhm *et al.* (2000 ▶); Jafarpour & Nolan (2001 ▶); Bourissou *et al.* (2000 ▶); Herrmann *et al.* (1996 ▶, 1997 ▶); Arduengo *et al.* (1991 ▶); Danopoulos *et al.* (2002 ▶); Dias & Jin (1994 ▶); Caballero *et al.* (2001 ▶); Thompson *et al.* (1999 ▶); Melaiye *et al.* (2005 ▶). For bond-length data, see: Allen *et al.* (1987 ▶). For a related structure, see: Haque *et al.* (2010 ▶).
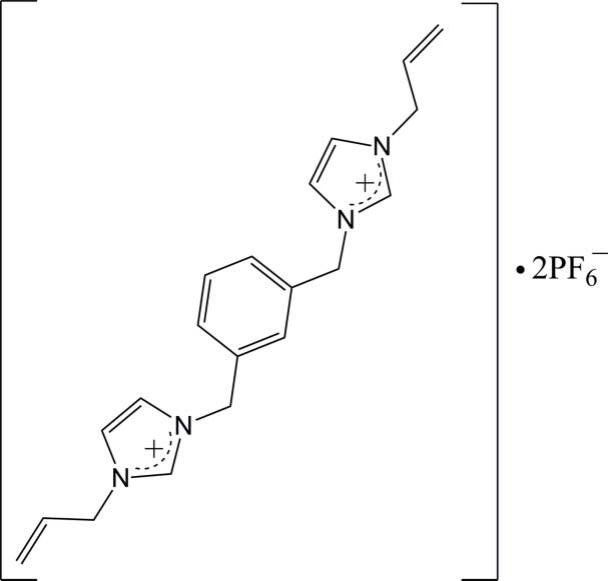


## Experimental

### 

#### Crystal data

C_20_H_24_N_4_^2+^·2PF_6_^−^
*M* *_r_* = 610.37
Monoclinic, 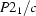

*a* = 9.8748 (4) Å
*b* = 9.9098 (3) Å
*c* = 26.124 (1) Å
β = 101.138 (2)°
*V* = 2508.27 (16) Å^3^
*Z* = 4
Mo *K*α radiation
μ = 0.28 mm^−1^
*T* = 296 K
0.25 × 0.20 × 0.20 mm

#### Data collection

Bruker SMART APEXII CCD area-detector diffractometer
Absorption correction: multi-scan (*SADABS*; Bruker, 2009 ▶) *T*_min_ = 0.921, *T*_max_ = 0.924
35601 measured reflections
5217 independent reflections
4324 reflections with *I* > 2σ(*I*)
*R*_int_ = 0.059

#### Refinement

*R*[*F* ^2^ > 2σ(*F* ^2^)] = 0.108
*wR*(*F* ^2^) = 0.201
*S* = 1.14
5217 reflections
431 parameters
H-atom parameters constrained
Δρ_max_ = 0.55 e Å^−3^
Δρ_min_ = −0.56 e Å^−3^

### 

Data collection: *APEX2* (Bruker, 2009 ▶); cell refinement: *SAINT* (Bruker, 2009 ▶); data reduction: *SAINT*; program(s) used to solve structure: *SHELXTL* (Sheldrick, 2008 ▶); program(s) used to refine structure: *SHELXTL*; molecular graphics: *SHELXTL*; software used to prepare material for publication: *SHELXTL* and *PLATON* (Spek, 2009 ▶).

## Supplementary Material

Crystal structure: contains datablocks global, I. DOI: 10.1107/S1600536810050683/rz2529sup1.cif

Structure factors: contains datablocks I. DOI: 10.1107/S1600536810050683/rz2529Isup2.hkl

Additional supplementary materials:  crystallographic information; 3D view; checkCIF report

## Figures and Tables

**Table 1:** Hydrogen-bond geometry (Å, °) *Cg*2 and *Cg*3 are the centroids of the N3*A*/N4*A*/C15*A*–C17*A* and N3*B*/N4*B*/C15*B*–C17*B* rings, respectively.

*D*—H⋯*A*	*D*—H	H⋯*A*	*D*⋯*A*	*D*—H⋯*A*
C3—H3*A*⋯F7*A*^i^	0.93	2.40	3.276 (6)	156
C5—H5*A*⋯F5^ii^	0.93	2.51	3.320 (6)	146
C8—H8*A*⋯F11*A*^iii^	0.93	2.27	3.107 (8)	150
C10—H10*A*⋯F7*A*	0.93	2.25	3.130 (6)	158
C16*A*—H16*A*⋯F6^iii^	0.93	2.48	3.407 (9)	172
C20*A*—H20*A*⋯*Cg*2	0.93	2.89	3.489 (11)	123
C20*B*—H20*C*⋯*Cg*3	0.93	2.84	3.44 (5)	124

Symmetry codes: (i) 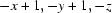; (ii) 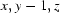; (iii) 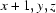.

**Fig. 1.**
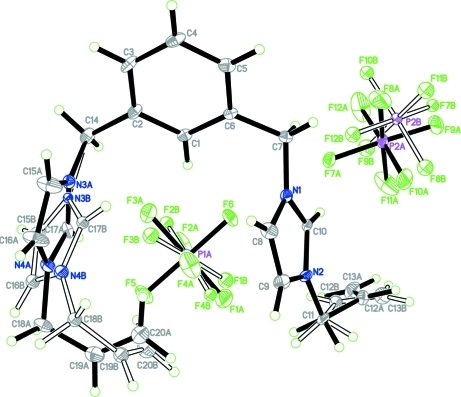
The molecular structure of the title compound showing 30% probability displacement ellipsoids for non-H atoms and the atom-numbering scheme. Both major and minor components of disorder are shown.

**Fig. 2.**
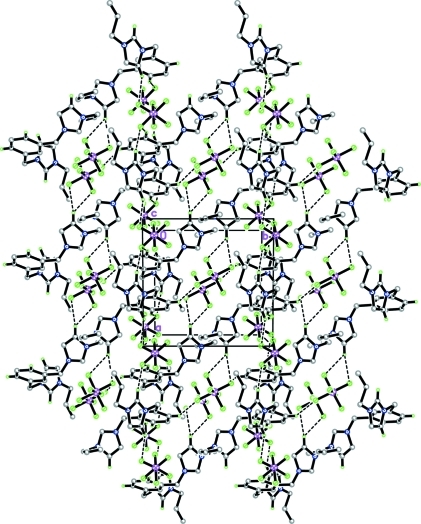
The crystal structure of the title compound, viewed along the c axis. H atoms not involved in hydrogen bonds (dashed lines) have been omitted for clarity. Only major component of disorder is shown.

## supplementary crystallographic information

### Comment

Carbenes are compounds possessing a neutral divalent carbon atom with six electrons on its valence shell. *N*-heterocyclic carbenes (NHCs) are cyclic carbenes that are usually derived from the deprotonation of imidazolium salts. NHCs are strong σ-donating ligands (Yang & Nolan, 2001; Böhm *et al.*, 2000) with negligible π -back bonding tendencies (Jafarpour &  Nolan, 2001; Bourissou *et al.*, 2000; Herrmann *et al.*, 1996). The isolation of crystalline stable free NHC by Arduengo (Arduengo *et al.*, 1991) provided information on their fundamental properties. Many researchers have since prepared free NHCs for use in other reactions (usually for metal complexation reactions). Danopoulos and co-worker (Danopoulos *et al.*, 2002) isolated a stable phosphine-functionalised carbene. Various compounds that contain more than one carbene centres are known (Dias & Jin, 1994; Caballero *et al.* , 2001). Carbene have the ability to form complexes with many metals (Herrmann *et al.*, 1997), of which transition metals are the most interesting due to their potentials for catalytic activity.  All transition metals have been complexed with NHC and the applications of compounds came to focus in many areas including the medical and biological sciences (Thompson *et al.*, 1999; Melaiye *et al.*, 2005).

In the title molecule (Fig. 1), the phenyl (C1-C6) ring is inclined at angles of 78.2 (3), 81.3 (4) and 73.9 (12)° with 1*H*-imidazol-3-ium (N1/N2/C8-C10, maximum deviation = 0.005 (5) Å at atom C10) ring, major component of 1*H*-imidazol-3-ium (N3A/N4A/C15A-C17A, maximum deviation = 0.008 (8) Å at atom N3A) ring and minor component of 1*H*-imidazol-3-ium (N3B/N4B/C15B-C17B, maximum deviation = 0.01 (3) Å at atoms N3B/C15B/C16B) ring, respectively. The ethene (C12/C13) and 3-allylimidazolium (N3/N4/C15-C20) moieties of the cation are disordered over two positions with refined site-occupancies of 0.664 (19) : 0.336 (19) and 0.784 (7) : 0.216 (7), respectively, whereas four fluorine atoms (F1A-F4A) of the hexafluoridophosphate (*A*) and a full hexafluoridophosphate (*B*) anion are disordered over two positions with refined site-occupancies of 0.764 (5) : 0.236 (5) and 0.847 (9) : 0.153 (9), respectively. Bond lengths (Allen *et al.*, 1987) and angles are within normal ranges and are comparable to a related structure (Haque *et al.*, 2010).

In the crystal packing (Fig. 2), the hexafluoridophosphate anions link the cations into two-dimensional networks parallel to (001) *via* intermolecular C3–H3A···F7A, C5–H5A···F5, C8–H8A···F11A, C10–H10A···F7A and C16A–H16A···F6 (Table 1) hydrogen bonds. π-π stacking interactions between the centroid of C1-C6 phenyl rings (Cg1), with Cg1···Cg1^i^ distance of 3.672 (3) Å [symmetry code: (i) 1-X, 1-Y, -Z] are observed. The crystal structure is further consilidated by C20A–H20A···Cg2, C20B–H20C···Cg3 (Table 1) interactions, where Cg2 and Cg3 are the centroid of N3A/N4A/C15A-C17A and N3B/N4B/C15B-C17B rings, respectively.

### Experimental

A mixture of imidazole (9.0 g, 130 mmol) and sodium hydroxide (5.0 g, 120 mmol) in DMSO (30 ml) was heated to 90 °C for 2 h. The mixture was cooled to room temperature and a solution of 1,3-bis(bromomethyl)benzene (15.0 g, 57 mmol) in DMSO (30 mL) was added, heated at 40 °C (1 h) and then poured into water (400 mL) followed by cooling in ice. Recrystallisation from methanol/water gives 1,3-bis(*N*-imidazole-1-yl methyl)benzene (1) as a white solid (9.0 g, 75%). Further, a mixture of 1 (0.5 g, 2.1 mmol) and allyl bromide (0.5 g, 4.2 mmol) in acetonitrile (30 mL) was refluxed at 90 °C for 24 h. The solvent was removed under reduced pressure to give 2.2Br as a pale-brown oil which was then reacted with KPF_6_ (0.22 g, 1.2 mmol) in 20 ml of methanol to yield the title compound. Recrystallisation from acetonitrile gives title compound as colourless solid. Yield: 0.4 g (60%). Crystals suitable for x-ray diffraction studies were obtained by slow evaporation of the salt solution in acetonitrile at room temperature.

### Refinement

All H atoms were positioned geometrically and refined using a riding model with C–H = 0.93-0.97 Å and *U*_iso_(H) = 1.2 *U*_eq_(C). The highest residual electron density peak is located at 1.09 Å from P2B and the deepest hole is located at 0.76 Å from F10A. The ethene (C12/C13) and the 3-allylimidazolium (N3/N4/C15-C20) moieties of the cation are disordered over two positions with refined site-occupancies of 0.664 (19):0.336 (19) and 0.784 (7):0.216 (7), respectively, whereas four fluorine atoms (F1A-F4A) of the hexafluoridophosphate (*A*) and a full hexafluoridophosphate (*B*) anion are disordered over two positions with refined site-occupancies of 0.764 (5):0.236 (5) and 0.847 (9): 0.153 (9), respectively. The minor components of disorder were refined isotropically. The rather large R-values are due to the tremendous amount of disorder in the crystal structure.

### Crystal data

**Table:**

C_20_H_24_N_4_^2+^·2PF_6_^−^	*F*(000) = 1240
*M**_r_* = 610.37	*D*_x_ = 1.616 Mg m^−^^3^
Monoclinic, *P*2_1_/*c*	Mo *K*α radiation, λ = 0.71073 Å
Hall symbol: -P 2ybc	Cell parameters from 9952 reflections
*a* = 9.8748 (4) Å	θ = 2.6–29.8°
*b* = 9.9098 (3) Å	µ = 0.28 mm^−^^1^
*c* = 26.124 (1) Å	*T* = 296 K
β = 101.138 (2)°	Block, colourless
*V* = 2508.27 (16) Å^3^	0.25 × 0.20 × 0.20 mm
*Z* = 4	

### Data collection

**Table:**

Bruker SMART APEXII CCD area-detector diffractometer	5217 independent reflections
Radiation source: fine-focus sealed tube	4324 reflections with *I* > 2σ(*I*)
graphite	*R*_int_ = 0.059
φ and ω scans	θ_max_ = 26.5°, θ_min_ = 2.1°
Absorption correction: multi-scan (*SADABS*; Bruker, 2009)	*h* = −12→12
*T*_min_ = 0.921, *T*_max_ = 0.924	*k* = −12→12
35601 measured reflections	*l* = −32→32

### Refinement

**Table:**

Refinement on *F*^2^	Primary atom site location: structure-invariant direct methods
Least-squares matrix: full	Secondary atom site location: difference Fourier map
*R*[*F*^2^ > 2σ(*F*^2^)] = 0.108	Hydrogen site location: inferred from neighbouring sites
*wR*(*F*^2^) = 0.201	H-atom parameters constrained
*S* = 1.14	*w* = 1/[σ^2^(*F*_o_^2^) + (0.*P*)^2^ + 16.2482*P*] where *P* = (*F*_o_^2^ + 2*F*_c_^2^)/3
5217 reflections	(Δ/σ)_max_ < 0.001
431 parameters	Δρ_max_ = 0.55 e Å^−^^3^
0 restraints	Δρ_min_ = −0.56 e Å^−^^3^

### Special details

**Table:**

Geometry. All esds (except the esd in the dihedral angle between two l.s. planes) are estimated using the full covariance matrix. The cell esds are taken into account individually in the estimation of esds in distances, angles and torsion angles; correlations between esds in cell parameters are only used when they are defined by crystal symmetry. An approximate (isotropic) treatment of cell esds is used for estimating esds involving l.s. planes.
Refinement. Refinement of F^2^ against ALL reflections. The weighted R-factor wR and goodness of fit S are based on F^2^, conventional R-factors R are based on F, with F set to zero for negative F^2^. The threshold expression of F^2^ > 2sigma(F^2^) is used only for calculating R-factors(gt) etc. and is not relevant to the choice of reflections for refinement. R-factors based on F^2^ are statistically about twice as large as those based on F, and R- factors based on ALL data will be even larger.

### Fractional atomic coordinates and isotropic or equivalent isotropic displacement parameters (Å^2^)

**Table:**

	*x*	*y*	*z*	*U*_iso_*/*U*_eq_	Occ. (<1)
N1	0.5923 (4)	0.5239 (4)	0.16321 (15)	0.0249 (9)	
N2	0.5777 (4)	0.6625 (4)	0.22597 (14)	0.0263 (9)	
C1	0.6305 (5)	0.5745 (5)	0.05490 (18)	0.0246 (10)	
H1A	0.5974	0.6492	0.0702	0.030*	
C2	0.6899 (5)	0.5907 (5)	0.01137 (18)	0.0296 (11)	
C3	0.7363 (6)	0.4786 (6)	−0.01166 (19)	0.0371 (13)	
H3A	0.7751	0.4891	−0.0411	0.044*	
C4	0.7257 (5)	0.3512 (6)	0.0086 (2)	0.0364 (13)	
H4A	0.7570	0.2765	−0.0072	0.044*	
C5	0.6685 (5)	0.3349 (5)	0.0524 (2)	0.0311 (11)	
H5A	0.6620	0.2494	0.0663	0.037*	
C6	0.6204 (5)	0.4472 (5)	0.07570 (18)	0.0261 (10)	
C7	0.5504 (6)	0.4246 (6)	0.1218 (2)	0.0381 (13)	
H7A	0.4512	0.4292	0.1100	0.046*	
H7B	0.5728	0.3349	0.1357	0.046*	
C8	0.7251 (5)	0.5590 (6)	0.1867 (2)	0.0376 (13)	
H8A	0.8062	0.5288	0.1774	0.045*	
C9	0.7151 (5)	0.6452 (6)	0.2256 (2)	0.0359 (13)	
H9A	0.7882	0.6858	0.2481	0.043*	
C10	0.5055 (5)	0.5895 (5)	0.18780 (16)	0.0224 (10)	
H10A	0.4097	0.5848	0.1794	0.027*	
C11	0.5195 (6)	0.7522 (6)	0.26188 (19)	0.0385 (14)	
H11A	0.5179	0.8445	0.2494	0.046*	0.664 (19)
H11B	0.5774	0.7490	0.2964	0.046*	0.664 (19)
H11C	0.5614	0.8395	0.2615	0.046*	0.336 (19)
H11D	0.5441	0.7173	0.2967	0.046*	0.336 (19)
C12A	0.3691 (10)	0.7065 (12)	0.2651 (4)	0.039 (2)	0.664 (19)
H12A	0.3593	0.6279	0.2835	0.046*	0.664 (19)
C13A	0.2577 (11)	0.7700 (13)	0.2440 (4)	0.055 (4)	0.664 (19)
H13A	0.2634	0.8489	0.2253	0.066*	0.664 (19)
H13B	0.1720	0.7367	0.2476	0.066*	0.664 (19)
C12B	0.3817 (18)	0.766 (2)	0.2496 (7)	0.031 (5)*	0.336 (19)
H12B	0.3441	0.8262	0.2233	0.037*	0.336 (19)
C13B	0.298 (3)	0.697 (2)	0.2737 (9)	0.050 (7)*	0.336 (19)
H13C	0.3340	0.6370	0.3000	0.061*	0.336 (19)
H13D	0.2030	0.7094	0.2642	0.061*	0.336 (19)
C14	0.7016 (6)	0.7302 (6)	−0.01020 (19)	0.0362 (13)	
H14A	0.7142	0.7244	−0.0460	0.043*	
H14B	0.6177	0.7806	−0.0097	0.043*	
N3A	0.8163 (9)	0.7969 (9)	0.0208 (3)	0.028 (2)	0.784 (7)
N4A	0.9335 (6)	0.9437 (8)	0.0716 (2)	0.0304 (13)	0.784 (7)
C15A	0.9519 (7)	0.7540 (10)	0.0311 (4)	0.064 (3)	0.784 (7)
H15A	0.9869	0.6761	0.0186	0.077*	0.784 (7)
C16A	1.0226 (8)	0.8469 (9)	0.0623 (4)	0.061 (3)	0.784 (7)
H16A	1.1170	0.8454	0.0756	0.073*	0.784 (7)
C17A	0.8076 (7)	0.9105 (8)	0.0463 (3)	0.0297 (16)	0.784 (7)
H17A	0.7272	0.9592	0.0465	0.036*	0.784 (7)
C18A	0.9690 (7)	1.0591 (7)	0.1078 (3)	0.0380 (17)	0.784 (7)
H18A	0.9070	1.1336	0.0962	0.046*	0.784 (7)
H18B	1.0623	1.0886	0.1072	0.046*	0.784 (7)
C19A	0.9587 (8)	1.0219 (8)	0.1620 (3)	0.046 (2)	0.784 (7)
H19A	0.9747	1.0913	0.1864	0.056*	0.784 (7)
C20A	0.9300 (15)	0.9046 (13)	0.1798 (4)	0.074 (4)	0.784 (7)
H20A	0.9128	0.8310	0.1574	0.089*	0.784 (7)
H20B	0.9266	0.8944	0.2150	0.089*	0.784 (7)
N3B	0.815 (3)	0.834 (4)	0.0292 (13)	0.014 (7)*	0.216 (7)
N4B	0.981 (2)	0.896 (2)	0.0929 (9)	0.028 (5)*	0.216 (7)
C15B	0.833 (3)	0.969 (3)	0.0291 (12)	0.039 (7)*	0.216 (7)
H15B	0.7829	1.0269	0.0045	0.047*	0.216 (7)
C16B	0.933 (3)	1.011 (3)	0.0687 (11)	0.029 (7)*	0.216 (7)
H16B	0.9612	1.0991	0.0774	0.034*	0.216 (7)
C17B	0.913 (2)	0.792 (2)	0.0705 (8)	0.021 (5)*	0.216 (7)
H17B	0.9292	0.7032	0.0810	0.025*	0.216 (7)
C18B	1.079 (2)	0.897 (2)	0.1437 (8)	0.027 (5)*	0.216 (7)
H18C	1.1247	0.8105	0.1488	0.032*	0.216 (7)
H18D	1.1488	0.9652	0.1423	0.032*	0.216 (7)
C19B	1.015 (3)	0.925 (3)	0.1883 (10)	0.031 (6)*	0.216 (7)
H19B	1.0729	0.9232	0.2209	0.037*	0.216 (7)
C20B	0.883 (4)	0.952 (4)	0.1874 (18)	0.056 (12)*	0.216 (7)
H20C	0.8213	0.9552	0.1557	0.067*	0.216 (7)
H20D	0.8531	0.9685	0.2184	0.067*	0.216 (7)
P1A	0.44160 (14)	0.94789 (14)	0.10748 (6)	0.0324 (3)	
F1A	0.4146 (8)	0.9819 (5)	0.1629 (2)	0.086 (2)	0.847 (9)
F2A	0.3010 (4)	1.0150 (5)	0.0787 (3)	0.073 (2)	0.847 (9)
F3A	0.4734 (6)	0.9122 (5)	0.0510 (2)	0.0663 (17)	0.847 (9)
F4A	0.5829 (5)	0.8749 (6)	0.1327 (2)	0.076 (2)	0.847 (9)
F5	0.5155 (4)	1.0898 (4)	0.10670 (16)	0.0693 (12)	
F6	0.3674 (3)	0.8058 (3)	0.10771 (14)	0.0463 (9)	
F1B	0.318 (3)	0.993 (3)	0.1411 (10)	0.045 (7)*	0.153 (9)
F2B	0.343 (3)	0.976 (2)	0.0549 (8)	0.037 (6)*	0.153 (9)
F3B	0.556 (3)	0.908 (2)	0.0792 (10)	0.045 (7)*	0.153 (9)
F4B	0.539 (2)	0.926 (2)	0.1628 (7)	0.026 (5)*	0.153 (9)
P2A	0.10000 (19)	0.4426 (3)	0.14929 (7)	0.0309 (6)	0.764 (5)
F7A	0.2000 (4)	0.5490 (5)	0.12970 (15)	0.0399 (12)	0.764 (5)
F8A	0.1804 (6)	0.3256 (6)	0.1277 (3)	0.079 (2)	0.764 (5)
F9A	0.0039 (8)	0.3374 (6)	0.1711 (3)	0.081 (2)	0.764 (5)
F10A	0.2148 (7)	0.4338 (6)	0.20314 (19)	0.0755 (19)	0.764 (5)
F11A	0.0331 (6)	0.5585 (6)	0.1761 (4)	0.099 (3)	0.764 (5)
F12A	−0.0023 (7)	0.4527 (10)	0.0967 (3)	0.127 (4)	0.764 (5)
P2B	0.0850 (6)	0.3710 (9)	0.1433 (2)	0.0242 (17)*	0.236 (5)
F7B	−0.0640 (14)	0.3262 (15)	0.1484 (5)	0.030 (3)*	0.236 (5)
F8B	0.1292 (17)	0.4035 (17)	0.2036 (6)	0.046 (4)*	0.236 (5)
F9B	0.0238 (18)	0.5234 (19)	0.1331 (7)	0.055 (5)*	0.236 (5)
F10B	0.0506 (14)	0.3422 (15)	0.0841 (5)	0.034 (3)*	0.236 (5)
F11B	0.1506 (15)	0.2290 (16)	0.1537 (6)	0.047 (4)*	0.236 (5)
F12B	0.2312 (14)	0.4321 (16)	0.1348 (5)	0.041 (4)*	0.236 (5)

### Atomic displacement parameters (Å^2^)

**Table:**

	*U*^11^	*U*^22^	*U*^33^	*U*^12^	*U*^13^	*U*^23^
N1	0.030 (2)	0.022 (2)	0.0226 (19)	0.0010 (17)	0.0056 (16)	0.0028 (16)
N2	0.034 (2)	0.028 (2)	0.0154 (18)	0.0004 (18)	0.0001 (16)	0.0012 (16)
C1	0.025 (2)	0.023 (3)	0.026 (2)	0.0111 (19)	0.0041 (18)	−0.001 (2)
C2	0.034 (3)	0.036 (3)	0.017 (2)	0.012 (2)	0.0016 (19)	0.005 (2)
C3	0.042 (3)	0.052 (4)	0.019 (2)	0.014 (3)	0.011 (2)	−0.001 (2)
C4	0.037 (3)	0.044 (3)	0.026 (3)	0.021 (3)	0.001 (2)	−0.008 (2)
C5	0.034 (3)	0.024 (3)	0.032 (3)	0.010 (2)	−0.002 (2)	0.001 (2)
C6	0.029 (2)	0.027 (3)	0.022 (2)	0.003 (2)	0.0029 (18)	−0.005 (2)
C7	0.055 (3)	0.028 (3)	0.035 (3)	−0.013 (3)	0.020 (3)	−0.010 (2)
C8	0.027 (3)	0.039 (3)	0.046 (3)	0.001 (2)	0.006 (2)	−0.002 (3)
C9	0.030 (3)	0.044 (3)	0.028 (3)	0.001 (2)	−0.007 (2)	0.003 (2)
C10	0.029 (2)	0.024 (2)	0.015 (2)	−0.0006 (19)	0.0050 (17)	0.0024 (18)
C11	0.066 (4)	0.033 (3)	0.020 (3)	−0.005 (3)	0.018 (3)	−0.007 (2)
C12A	0.046 (6)	0.038 (6)	0.035 (5)	−0.007 (4)	0.017 (4)	−0.001 (4)
C13A	0.053 (7)	0.077 (8)	0.038 (6)	−0.005 (6)	0.016 (5)	−0.005 (5)
C14	0.042 (3)	0.043 (3)	0.021 (2)	0.005 (3)	0.000 (2)	0.010 (2)
N3A	0.034 (4)	0.025 (5)	0.026 (4)	0.000 (3)	0.009 (3)	0.003 (3)
N4A	0.030 (3)	0.025 (4)	0.037 (3)	−0.004 (3)	0.007 (3)	−0.003 (3)
C15A	0.021 (4)	0.065 (6)	0.109 (8)	−0.005 (4)	0.021 (4)	−0.049 (6)
C16A	0.022 (4)	0.058 (6)	0.099 (7)	−0.003 (4)	0.006 (4)	−0.044 (5)
C17A	0.024 (3)	0.026 (4)	0.041 (4)	−0.004 (3)	0.009 (3)	0.009 (4)
C18A	0.038 (4)	0.023 (4)	0.050 (4)	−0.003 (3)	0.001 (3)	−0.009 (3)
C19A	0.040 (4)	0.045 (5)	0.053 (5)	−0.004 (4)	0.007 (4)	−0.011 (4)
C20A	0.107 (11)	0.076 (9)	0.041 (5)	−0.039 (8)	0.016 (6)	−0.004 (5)
P1A	0.0294 (7)	0.0260 (7)	0.0402 (8)	−0.0003 (6)	0.0022 (6)	0.0082 (6)
F1A	0.150 (7)	0.056 (3)	0.067 (4)	−0.014 (4)	0.055 (4)	−0.009 (3)
F2A	0.031 (2)	0.048 (3)	0.137 (6)	0.011 (2)	0.007 (3)	0.046 (3)
F3A	0.079 (4)	0.070 (3)	0.056 (3)	−0.004 (3)	0.028 (3)	0.002 (3)
F4A	0.034 (2)	0.076 (4)	0.105 (5)	0.000 (2)	−0.015 (3)	0.037 (3)
F5	0.084 (3)	0.046 (2)	0.068 (3)	−0.031 (2)	−0.008 (2)	0.015 (2)
F6	0.0441 (19)	0.0322 (18)	0.058 (2)	−0.0076 (15)	−0.0021 (16)	0.0128 (16)
P2A	0.0319 (10)	0.0293 (14)	0.0350 (10)	0.0034 (9)	0.0153 (7)	0.0049 (9)
F7A	0.035 (2)	0.055 (3)	0.031 (2)	−0.010 (2)	0.0088 (17)	0.003 (2)
F8A	0.083 (4)	0.054 (4)	0.113 (5)	0.005 (3)	0.051 (4)	−0.025 (4)
F9A	0.104 (5)	0.070 (4)	0.087 (5)	−0.041 (4)	0.067 (4)	−0.015 (4)
F10A	0.097 (5)	0.082 (4)	0.039 (3)	−0.017 (4)	−0.008 (3)	0.025 (3)
F11A	0.057 (3)	0.063 (4)	0.198 (8)	−0.008 (3)	0.082 (4)	−0.032 (5)
F12A	0.086 (5)	0.165 (8)	0.095 (5)	−0.078 (5)	−0.070 (4)	0.070 (6)

### Geometric parameters (Å, °)

**Table:**

N1—C10	1.335 (6)	C15A—H15A	0.9300
N1—C8	1.381 (6)	C16A—H16A	0.9300
N1—C7	1.462 (6)	C17A—H17A	0.9300
N2—C10	1.323 (6)	C18A—C19A	1.487 (11)
N2—C9	1.370 (7)	C18A—H18A	0.9700
N2—C11	1.486 (6)	C18A—H18B	0.9700
C1—C6	1.385 (7)	C19A—C20A	1.303 (14)
C1—C2	1.386 (6)	C19A—H19A	0.9300
C1—H1A	0.9300	C20A—H20A	0.9300
C2—C3	1.383 (7)	C20A—H20B	0.9300
C2—C14	1.506 (7)	N3B—C15B	1.35 (5)
C3—C4	1.380 (8)	N3B—C17B	1.37 (4)
C3—H3A	0.9300	N4B—C17B	1.30 (3)
C4—C5	1.379 (7)	N4B—C16B	1.35 (4)
C4—H4A	0.9300	N4B—C18B	1.48 (3)
C5—C6	1.395 (7)	C15B—C16B	1.35 (4)
C5—H5A	0.9300	C15B—H15B	0.9300
C6—C7	1.517 (7)	C16B—H16B	0.9300
C7—H7A	0.9700	C17B—H17B	0.9300
C7—H7B	0.9700	C18B—C19B	1.45 (3)
C8—C9	1.344 (8)	C18B—H18C	0.9700
C8—H8A	0.9300	C18B—H18D	0.9700
C9—H9A	0.9300	C19B—C20B	1.32 (5)
C10—H10A	0.9300	C19B—H19B	0.9300
C11—C12B	1.343 (18)	C20B—H20C	0.9300
C11—C12A	1.570 (11)	C20B—H20D	0.9300
C11—H11A	0.9700	P1A—F3B	1.52 (2)
C11—H11B	0.9700	P1A—F2B	1.55 (2)
C11—H11C	0.9600	P1A—F1A	1.558 (5)
C11—H11D	0.9601	P1A—F5	1.586 (4)
C12A—C13A	1.294 (17)	P1A—F4B	1.587 (18)
C12A—H12A	0.9300	P1A—F6	1.588 (3)
C13A—H13A	0.9300	P1A—F2A	1.592 (4)
C13A—H13B	0.9300	P1A—F4A	1.597 (5)
C12B—C13B	1.32 (3)	P1A—F3A	1.606 (5)
C12B—H12B	0.9300	P1A—F1B	1.69 (2)
C13B—H13C	0.9300	P2A—F12A	1.543 (5)
C13B—H13D	0.9300	P2A—F11A	1.557 (6)
C14—N3A	1.422 (10)	P2A—F8A	1.570 (6)
C14—N3B	1.71 (3)	P2A—F9A	1.587 (5)
C14—H14A	0.9700	P2A—F7A	1.595 (4)
C14—H14B	0.9700	P2A—F10A	1.629 (5)
N3A—C17A	1.319 (11)	P2B—F10B	1.543 (14)
N3A—C15A	1.380 (11)	P2B—F11B	1.552 (17)
N4A—C17A	1.332 (9)	P2B—F7B	1.567 (15)
N4A—C16A	1.354 (10)	P2B—F8B	1.586 (17)
N4A—C18A	1.482 (9)	P2B—F12B	1.620 (15)
C15A—C16A	1.335 (11)	P2B—F9B	1.628 (19)
			
C10—N1—C8	107.8 (4)	C19A—C20A—H20A	120.0
C10—N1—C7	124.6 (4)	C19A—C20A—H20B	120.0
C8—N1—C7	127.4 (4)	H20A—C20A—H20B	120.0
C10—N2—C9	108.4 (4)	C15B—N3B—C17B	103 (3)
C10—N2—C11	125.8 (4)	C15B—N3B—C14	132 (3)
C9—N2—C11	125.7 (4)	C17B—N3B—C14	125 (3)
C6—C1—C2	120.1 (4)	C17B—N4B—C16B	111 (2)
C6—C1—H1A	120.0	C17B—N4B—C18B	127 (2)
C2—C1—H1A	120.0	C16B—N4B—C18B	121 (2)
C3—C2—C1	119.5 (5)	C16B—C15B—N3B	112 (3)
C3—C2—C14	121.3 (5)	C16B—C15B—H15B	123.8
C1—C2—C14	119.2 (5)	N3B—C15B—H15B	123.8
C4—C3—C2	120.8 (5)	C15B—C16B—N4B	104 (3)
C4—C3—H3A	119.6	C15B—C16B—H16B	128.1
C2—C3—H3A	119.6	N4B—C16B—H16B	128.1
C5—C4—C3	119.9 (5)	N4B—C17B—N3B	110 (2)
C5—C4—H4A	120.0	N4B—C17B—H17B	125.0
C3—C4—H4A	120.0	N3B—C17B—H17B	125.0
C4—C5—C6	119.8 (5)	C19B—C18B—N4B	114 (2)
C4—C5—H5A	120.1	C19B—C18B—H18C	108.7
C6—C5—H5A	120.1	N4B—C18B—H18C	108.7
C1—C6—C5	119.9 (4)	C19B—C18B—H18D	108.7
C1—C6—C7	121.6 (4)	N4B—C18B—H18D	108.7
C5—C6—C7	118.3 (5)	H18C—C18B—H18D	107.6
N1—C7—C6	112.4 (4)	C20B—C19B—C18B	127 (3)
N1—C7—H7A	109.1	C20B—C19B—H19B	116.6
C6—C7—H7A	109.1	C18B—C19B—H19B	116.6
N1—C7—H7B	109.1	C19B—C20B—H20C	120.0
C6—C7—H7B	109.1	C19B—C20B—H20D	120.0
H7A—C7—H7B	107.9	H20C—C20B—H20D	120.0
C9—C8—N1	107.1 (5)	F3B—P1A—F2B	90.9 (13)
C9—C8—H8A	126.4	F3B—P1A—F1A	141.7 (10)
N1—C8—H8A	126.4	F2B—P1A—F1A	126.4 (10)
C8—C9—N2	107.6 (5)	F3B—P1A—F5	80.5 (9)
C8—C9—H9A	126.2	F2B—P1A—F5	92.6 (9)
N2—C9—H9A	126.2	F1A—P1A—F5	88.9 (3)
N2—C10—N1	109.1 (4)	F3B—P1A—F4B	91.7 (12)
N2—C10—H10A	125.5	F2B—P1A—F4B	176.8 (12)
N1—C10—H10A	125.5	F1A—P1A—F4B	50.7 (7)
C12B—C11—N2	114.1 (8)	F5—P1A—F4B	86.0 (7)
N2—C11—C12A	110.2 (5)	F3B—P1A—F6	99.3 (9)
C12B—C11—H11A	82.7	F2B—P1A—F6	87.0 (8)
N2—C11—H11A	109.6	F1A—P1A—F6	91.5 (3)
C12A—C11—H11A	109.6	F5—P1A—F6	179.5 (2)
C12B—C11—H11B	127.9	F4B—P1A—F6	94.4 (7)
N2—C11—H11B	109.6	F3B—P1A—F2A	122.9 (10)
C12A—C11—H11B	109.6	F1A—P1A—F2A	93.3 (4)
H11A—C11—H11B	108.1	F5—P1A—F2A	89.1 (2)
C12B—C11—H11C	108.9	F4B—P1A—F2A	143.7 (8)
N2—C11—H11C	108.1	F6—P1A—F2A	90.7 (2)
C12A—C11—H11C	132.5	F3B—P1A—F4A	53.8 (10)
H11B—C11—H11C	82.0	F2B—P1A—F4A	142.9 (10)
C12B—C11—H11D	108.9	F1A—P1A—F4A	90.4 (4)
N2—C11—H11D	109.0	F5—P1A—F4A	92.3 (3)
C12A—C11—H11D	85.1	F6—P1A—F4A	87.9 (2)
H11A—C11—H11D	130.0	F2A—P1A—F4A	176.0 (4)
H11C—C11—H11D	107.7	F2B—P1A—F3A	54.9 (9)
C13A—C12A—C11	124.9 (11)	F1A—P1A—F3A	178.6 (4)
C13A—C12A—H12A	117.6	F5—P1A—F3A	90.7 (3)
C11—C12A—H12A	117.6	F4B—P1A—F3A	127.9 (8)
C12A—C13A—H13A	120.0	F6—P1A—F3A	88.9 (2)
C12A—C13A—H13B	120.0	F2A—P1A—F3A	88.0 (3)
H13A—C13A—H13B	120.0	F4A—P1A—F3A	88.2 (3)
C13B—C12B—C11	122 (2)	F3B—P1A—F1B	177.8 (13)
C13B—C12B—H12B	119.0	F2B—P1A—F1B	91.2 (12)
C11—C12B—H12B	119.0	F5—P1A—F1B	98.6 (9)
C12B—C13B—H13C	120.0	F4B—P1A—F1B	86.1 (11)
C12B—C13B—H13D	120.0	F6—P1A—F1B	81.7 (9)
H13C—C13B—H13D	120.0	F2A—P1A—F1B	59.1 (9)
N3A—C14—C2	108.9 (5)	F4A—P1A—F1B	124.3 (9)
C2—C14—N3B	114.6 (12)	F3A—P1A—F1B	145.4 (9)
N3A—C14—H14A	109.9	F12A—P2A—F11A	95.2 (5)
C2—C14—H14A	109.9	F12A—P2A—F8A	91.0 (5)
N3B—C14—H14A	115.0	F11A—P2A—F8A	173.8 (4)
N3A—C14—H14B	109.9	F12A—P2A—F9A	91.6 (4)
C2—C14—H14B	109.9	F11A—P2A—F9A	89.3 (3)
N3B—C14—H14B	98.2	F8A—P2A—F9A	91.3 (4)
H14A—C14—H14B	108.3	F12A—P2A—F7A	90.6 (3)
C17A—N3A—C15A	108.7 (7)	F11A—P2A—F7A	90.0 (3)
C17A—N3A—C14	124.2 (7)	F8A—P2A—F7A	89.2 (3)
C15A—N3A—C14	127.1 (8)	F9A—P2A—F7A	177.7 (3)
C17A—N4A—C16A	108.3 (7)	F12A—P2A—F10A	176.9 (4)
C17A—N4A—C18A	126.2 (7)	F11A—P2A—F10A	86.3 (4)
C16A—N4A—C18A	125.3 (6)	F8A—P2A—F10A	87.5 (4)
C16A—C15A—N3A	106.2 (7)	F9A—P2A—F10A	91.1 (4)
C16A—C15A—H15A	126.9	F7A—P2A—F10A	86.7 (3)
N3A—C15A—H15A	126.9	F10B—P2B—F11B	90.6 (9)
C15A—C16A—N4A	108.4 (7)	F10B—P2B—F7B	90.3 (8)
C15A—C16A—H16A	125.8	F11B—P2B—F7B	95.5 (9)
N4A—C16A—H16A	125.8	F10B—P2B—F8B	176.6 (9)
N3A—C17A—N4A	108.4 (7)	F11B—P2B—F8B	89.1 (8)
N3A—C17A—H17A	125.8	F7B—P2B—F8B	93.1 (8)
N4A—C17A—H17A	125.8	F10B—P2B—F12B	87.8 (7)
N4A—C18A—C19A	111.5 (6)	F11B—P2B—F12B	90.6 (8)
N4A—C18A—H18A	109.3	F7B—P2B—F12B	173.7 (9)
C19A—C18A—H18A	109.3	F8B—P2B—F12B	88.8 (8)
N4A—C18A—H18B	109.3	F10B—P2B—F9B	90.3 (9)
C19A—C18A—H18B	109.3	F11B—P2B—F9B	177.1 (10)
H18A—C18A—H18B	108.0	F7B—P2B—F9B	87.2 (9)
C20A—C19A—C18A	128.4 (8)	F8B—P2B—F9B	89.8 (10)
C20A—C19A—H19A	115.8	F12B—P2B—F9B	86.8 (9)
C18A—C19A—H19A	115.8		
			
C6—C1—C2—C3	1.3 (7)	C3—C2—C14—N3B	−112.3 (13)
C6—C1—C2—C14	−179.2 (5)	C1—C2—C14—N3B	68.2 (14)
C1—C2—C3—C4	−0.9 (8)	C2—C14—N3A—C17A	−120.4 (7)
C14—C2—C3—C4	179.7 (5)	N3B—C14—N3A—C17A	1(6)
C2—C3—C4—C5	−0.1 (8)	C2—C14—N3A—C15A	57.2 (10)
C3—C4—C5—C6	0.6 (8)	C17A—N3A—C15A—C16A	−1.1 (11)
C2—C1—C6—C5	−0.9 (7)	C14—N3A—C15A—C16A	−179.1 (8)
C2—C1—C6—C7	−177.2 (5)	N3A—C15A—C16A—N4A	0.5 (12)
C4—C5—C6—C1	−0.1 (7)	C17A—N4A—C16A—C15A	0.4 (12)
C4—C5—C6—C7	176.4 (5)	C18A—N4A—C16A—C15A	174.9 (8)
C10—N1—C7—C6	133.8 (5)	C15A—N3A—C17A—N4A	1.4 (9)
C8—N1—C7—C6	−51.7 (7)	C14—N3A—C17A—N4A	179.4 (7)
C1—C6—C7—N1	−45.3 (7)	C16A—N4A—C17A—N3A	−1.1 (9)
C5—C6—C7—N1	138.3 (5)	C18A—N4A—C17A—N3A	−175.5 (7)
C10—N1—C8—C9	0.4 (6)	C17A—N4A—C18A—C19A	87.4 (10)
C7—N1—C8—C9	−174.8 (5)	C16A—N4A—C18A—C19A	−86.1 (10)
N1—C8—C9—N2	0.2 (6)	N4A—C18A—C19A—C20A	3.4 (14)
C10—N2—C9—C8	−0.7 (6)	N3A—C14—N3B—C15B	133 (9)
C11—N2—C9—C8	−178.5 (5)	C2—C14—N3B—C15B	−164 (3)
C9—N2—C10—N1	1.0 (5)	N3A—C14—N3B—C17B	−47 (6)
C11—N2—C10—N1	178.7 (4)	C2—C14—N3B—C17B	15 (3)
C8—N1—C10—N2	−0.9 (5)	C17B—N3B—C15B—C16B	−2(4)
C7—N1—C10—N2	174.5 (4)	C14—N3B—C15B—C16B	178 (3)
C10—N2—C11—C12B	−6.5 (13)	N3B—C15B—C16B—N4B	2(4)
C9—N2—C11—C12B	170.9 (12)	C17B—N4B—C16B—C15B	−2(3)
C10—N2—C11—C12A	23.7 (8)	C18B—N4B—C16B—C15B	−171 (2)
C9—N2—C11—C12A	−159.0 (6)	C16B—N4B—C17B—N3B	1(3)
C12B—C11—C12A—C13A	−3.5 (16)	C18B—N4B—C17B—N3B	169 (2)
N2—C11—C12A—C13A	−107.0 (9)	C15B—N3B—C17B—N4B	1(3)
N2—C11—C12B—C13B	99.6 (18)	C14—N3B—C17B—N4B	−179 (2)
C12A—C11—C12B—C13B	11.5 (14)	C17B—N4B—C18B—C19B	−90 (3)
C3—C2—C14—N3A	−101.3 (6)	C16B—N4B—C18B—C19B	78 (3)
C1—C2—C14—N3A	79.2 (7)	N4B—C18B—C19B—C20B	−2(4)

### Hydrogen-bond geometry (Å, °)

**Table:**

Cg2 and Cg3 are the centroids of the N3A/N4A/C15A–C17A and N3B/N4B/C15B–C17B rings, respectively.

**Table:**

*D*—H···*A*	*D*—H	H···*A*	*D*···*A*	*D*—H···*A*
C3—H3A···F7A^i^	0.93	2.40	3.276 (6)	156
C5—H5A···F5^ii^	0.93	2.51	3.320 (6)	146
C8—H8A···F11A^iii^	0.93	2.27	3.107 (8)	150
C10—H10A···F7A	0.93	2.25	3.130 (6)	158
C16A—H16A···F6^iii^	0.93	2.48	3.407 (9)	172
C20A—H20A···Cg2	0.93	2.89	3.489 (11)	123
C20B—H20C···Cg3	0.93	2.84	3.44 (5)	124

Symmetry codes: (i) −*x*+1, −*y*+1, −*z*; (ii) *x*, *y*−1, *z*; (iii) *x*+1, *y*, *z*.
